# The impact of clock timing on VDT visual search performance under time constraint

**DOI:** 10.3389/fpsyg.2024.1369920

**Published:** 2024-07-15

**Authors:** Jiabin Hu, Qun Chen, Danqiong Lu, Jingkang He

**Affiliations:** ^1^School of Art and Design, North China Institute of Science and Technology, Langfang, Hebei, China; ^2^School of Electronics and Information Engineering, North China Institute of Science and Technology, Langfang, Hebei, China

**Keywords:** clock timing, visual search, time constraint, time pressure, interface design, cognition

## Abstract

**Introduction:**

Conducting Visual Display Terminal (VDT) visual search tasks under time constraint has broad applications in fields such as security checks, medical diagnostics, and rescue operations. While excessive time pressure can impair performance, moderate time pressure can motivate individuals to complete tasks and increase productivity. Investigating the positive impact of time pressure on visual search tasks has become a crucial area of study. Clock timing plays a vital role in the visual interface, influencing the perception of time pressure and impacting visual search performance. However, existing research has paid little attention to the induction of time pressure and the impact of clock timing in VDT visual interfaces on visual search performance. Hence, the objective of this study is to investigate the impact of clock timing on VDT visual search performance under time constraint.

**Methods:**

The content of the experimental tasks was determined through a pilot experiment. The formal experiment was conducted in two phases over six sessions. Participants were tasked with locating the letter “E” embedded within the distractor letter “F,” displayed with a clock area above the interface. The first phase of experiments included conditions of no clock, 4-min clock timing, and 4-min countdown clock timing. In the second phase of the experiment, the clock display method was a countdown clock, with three experiments conducted featuring long time, medium time, and short time. Search speed and accuracy were used as primary performance evaluation metrics to examine the impact of clock timing methods and duration on visual search performance. Twenty-one undergraduate students participated in the formal experiment.

**Results:**

In the first phase of experiments, participants demonstrated significantly faster reaction times (RTs) in tasks where a clock display was present compared to tasks without (ANOVA, F(2, 60) = 4.588, *P* = 0.014). However, there were no significant differences in accuracy rates across different timing conditions (ANOVA, F(2, 60) = 0.146, *P* = 0.865), and no significant correlation between RTs and accuracy was found (Kendall’s *R* = 0.11, *P* = 0.914). During the second phase, RTs decreased significantly as time constraints became more stringent (ANOVA, F(2, 60) = 7.564, *P* < 0.05). Conversely, accuracy rates decreased significantly under shorter time constraints (ANOVA, F(2, 60) = 4.315, *P* < 0.05), with a negative correlation observed between RTs and accuracy (Kendall’s *R* = 0.220, *P* < 0.01).

**Discussion:**

Compared to conditions without clock displays, having clock displays significantly improved the speed of the visual search task, although the difference in accuracy was not statistically significant. In the context of shorter clock countdown limits, Shorter timing constraints resulted in faster search speeds but also led to reduced accuracy and increased fatigue. Overall, a correlation exists between search speed and accuracy in visual tasks, where higher speed often correlates with lower accuracy. These findings provide valuable insights into clock timing design for visual search interfaces under time pressure.

## 1 Introduction

Visual search via visual display terminals (VDTs) has a wide range of applications in areas such as security checks (Rieger et al., 2021), medical diagnostics (Hebert et al., 2020), and rescue operations (Li et al., 2022), however, these tasks are usually accompanied by a certain amount of time pressure. For example, in airport baggage security checks, staff members need to search X-ray images using VDT in a relatively short period of time to find potential contraband and maximize the search efficiency. Time pressure is usually determined by factors such as task attributes and affects the visual search performance of operators (Roberts, 2009). Tasks under significant time pressure are typically more demanding and prone to errors (Van der Vegt et al., 2020; Qu et al., 2022). However, time pressure can also have certain facilitating effects on individual task performance, motivating individuals to complete tasks and enhancing work efficiency (Ackerman and Lauterman, 2012; Rice and Trafimow, 2012). Investigating the positive impact of time pressure on visual search tasks has become a crucial area of study.

Clock timing is a pivotal component of the VDT visual search interface and is also a significant source of time pressure. However, existing research has paid little attention to the induction of time pressure and the impact of clock timing in VDT visual interfaces on visual search performance. Irwin et al. (2013) used a 12-min digital clock to induce time pressure, studying the impact of factors such as time pressure and tall man lettering on the visual search of drug names. Nowakowska et al. (2021) designed time constraints of 2 s (brief condition) and 60 s (long condition), requiring participants to respond within the specified time. Klapproth and Müller (2008) demonstrated that different time constraints can have a significant impact on human perception of time intervals, affecting both speed and accuracy. This shows that it is important to conduct an in-depth study of clock timing duration. Based on this, we investigate the impact of objective factors such as clock timing methods and various time constraints under the background of time pressure on VDT visual search task performance. It also aims to explore the underlying mechanisms to provide references for clock design in the visual interface under time pressure, thereby enhancing the efficiency of executing search tasks.

## 2 Theoretical background and research questions

### 2.1 Time perception and time pressure

Individuals perceive time through either a linear or cyclical perspective. The linear view conceptualizes time as a unidirectional path, where once time passes, it cannot be retrieved (Lightfoot and Lyra, 2000). Conversely, the cyclical perspective views time as a recurring process, cyclically returning to its starting point (Yamada and Kato, 2006). Temporal duration refers to the time interval or span, while time perception encompasses individuals’ judgments of time, spanning from milliseconds to several minutes (Grondin, 2010). The presence of clock timing can significantly refine temporal duration perception and affect one’s overall sense of time. In this study, employing a linear timing approach via a digital clock continuously adjusted participants’ estimations of time used, thus influencing the precision of temporal duration perception and subsequently impacting task efficiency.

The activation theory and the vitamin theory are representative theories of time pressure modeling. The activation theory posits that optimal task performance occurs only when time pressure reaches an optimal activation level. The relationship between time pressure and behavior often follows an inverted “U” curve (Baer and Oldham, 2006), although some studies propose a “J” or an inverted “J” curve (Song et al., 2022). The vitamin theory likens time pressure to vitamins, suggesting that excessive or insufficient time pressure can adversely affect human performance. Only appropriate levels of time pressure contribute positively to task completion (Warr, 2007). By integrating the perspectives of the activation theory and the vitamin theory, it becomes evident that suitable time pressure can enhance task performance.

In Lin and Jia’s (2023) study regarding the link between time pressure and risk decision-making, participants were tasked with completing a simple gambling task within time constraints of 1,000, 2,000, and 3,000 ms, representing high, medium, and low levels of time pressure, respectively. Weenig and Maarleveld (2002) categorized low time pressure as values below 1 standard deviation (SD) from the mean completion time, considering 50% of the mean task completion time as high time pressure. Similarly, other researchers utilized 1 SD above and below the mean completion time to represent low and high time pressure scenarios (Liao, 2023). Employing different time constraints to signify distinct levels of time pressure has become a prevalent approach, which aligns with the methodology applied in this study.

### 2.2 Methods for visual search experiments

Various scholars have proposed diverse methodologies for conducting visual search experiments. We conducted experiments with reference to these methods and paradigms. In the method of time pressure elicitation, Alqahtani et al. (2016) conducted an experiment where participants were actively encouraged to imagine themselves in a busy environment, studying the impact of time pressure on the diagnostic accuracy of doctors. Morrongiello et al. (2015) investigated the impact of time pressure on children and adults crossing the road through a constructed visual virtual environment. In terms of experimental methods for visual search in the context of time pressure, (Rajsic et al., 2015, 2018) devised an experimental task featuring eight letters (b/d/p/q) arranged in a circular formation at the screen center, with a single “p” among them. The letters were presented in red or blue, and participants were tasked with locating the letter “p.” Difficulty levels were manipulated by altering the proportion of red letters. In Sha et al.’s (2018) experiment, participants were directed to search for the target letter “E” amidst numerous distractor letters “F” by using faux X-ray images. Each image contained at most one letter “E,” prompting participants to press the “q” key if the letter “E” was not found and the “w” key if it was found. This methodology was similarly employed in Rieger and Manzey’s (2024), Hebert et al.’s (2020), Burgess et al.’s (2001) study. Wu and Wolfe’s (2022) experimental materials required participants to spot the letter “T” among various letters “L,” with the letters rotated at different angles. Consequently, visual search experiments often involve tasks necessitating the identification of a target letter from a set of visually similar letters. However, the above experimental paradigm has only one target at a time in the experimental task, and there is a certain amount of randomness and chance in the search. In terms of search strategy, systematic search and random search are two forms of user search (Starke and Baber, 2018). Random search has a certain degree of randomness, and if there is only one target in the target task, the randomness of the search is stronger. Therefore, in order to avoid the effect of random search on the experimental results, on the one hand, participants are required to perform the systematic search, and on the other hand, the number of targets in the design search task is not unique. Furthermore, the visual interface significantly influences search performance (Xu and Wang, 2022), with factors such as element shape, compactness, and layout in the horizontal and vertical dimensions significantly impacting visual search efficiency (Grobelny et al., 2005). Square icons and horizontal layouts have shown higher efficiency for visual search compared to vertical layouts (Michalski and Grobelny, 2008; Michalski et al., 2012).

Visual interfaces can be categorized into structured and unstructured regions based on content layout. Structured regions exhibit specific arrangements, leading individuals to adopt systematic search strategies within these regions. Given the uncertainty surrounding the number of search targets in most tasks, a structured design is implemented, compelling participants to employ systematic search strategies for individual searches.

Response Time (RT) and Accuracy (ACC) serve as primary assessment indicators for search performance (Moacdieh and Sarter, 2017). Kee (2017) provided an analysis of models like speed accuracy trade-off (SATO) and visual lobe-based models, delineating their advantages and constraints. Professions such as security screening and emergency rescue demand error-free performance, emphasizing high accuracy. Conversely, in sampling inspections, swift search speeds are essential. Additionally, employing eye-tracking devices to measure metrics like fixation points and counts has been instrumental in evaluating visual search performance (Tong et al., 2022).

### 2.3 Research questions

**FIGURE 1:**
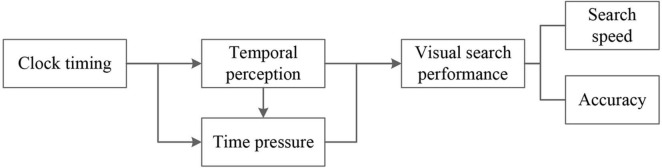
Theoretical model.

In summary, the theoretical model illustrated in Figure 1 highlights the influence of clock timing on individuals’ time perception, leading to the manifestation of time pressure and subsequently affecting visual search performance. The evaluation of visual search performance typically relies on two pivotal indicators: search speed and accuracy. While existing studies have predominantly delved into visual search performance under time pressure, they have often overlooked the significant role of clock timing display within the interface, its ability to induce time pressure, and its consequent impact on search performance. Consequently, there arises a need to thoroughly investigate the effects of timing methods and varying timing intervals within the visual search interface on overall search performance. Specific research inquiries encompass:

1. The presence of a clock timing display versus no clock timing may have an impact on performance on visual search tasks under time pressure. What are the effects?
2. What are the effects of clock timing with different time constraints on visual search task performance? What levels of clock timing favor performance levels?
3. What are the adverse consequences that emerge concerning visual search task performance when impacted by clock timing adjustments aimed at augmenting overall efficiency?

## 3 Experimental design and method

### 3.1 Content of the visual search task

The visual search task interface typically employs a 16:9 aspect ratio, which was adopted in this experiment as well, partitioning the interface into two sections: the time presentation segment and the search task content area. To balance the time display’s impact on participants’ task execution without overshadowing the search task, the time presentation was placed at the interface’s top, occupying no more than 10% of the interface width. Two forms of time presentation, a timer and a countdown timer, were utilized. As single search tasks usually last a few tens of seconds, presenting time in milliseconds could cause visual interference. Hence, the timer exhibited time in the “minutes:seconds” format.

For the search content design, following the previously mentioned paradigm, a 16 × 8 grid was generated, totaling 128 squares, with concealed gridlines. Each square contained either the letters “F” or “E,” where “F” represented distractors and “E” denoted the target. These letters underwent rotations of 90°, 180°, and 270° and mirror transformations, ensuring each letter occupied more than 60% of the square’s space, placed randomly. Memory can have an effect on mixed visual search (Wolfe, 2012), and to avoid this effect and the user’s randomized search strategy, pilot experiments were conducted to determine the stimulus content of the search task.

### 3.2 Pilot experiment

The presence of multiple target items ensures that participants can use a systematic search strategy to carry out the visual search task, avoiding the effects of memory on performance and the effects of fatigue on experimental results during the experiment. A pilot experiment is needed to determine the range of the number of target items in the search task and the duration of the experiment.

Five undergraduate students from a specific university participated in this study. All participants were right-handed and had a visual acuity of 5.0 or higher, either corrected or uncorrected. Before commencing the experiment, all participants volunteered and were fully briefed on the nature of the visual search task. At the end of the experiment, participants received a small remuneration. The primary experimental equipment included a computer with a 23-inch screen, with a resolution of 1,920 × 1,080 pixels and a refresh rate of 85 Hz. The experimental environment was carefully maintained with optimal temperature, moderate humidity, suitable lighting, and a quiet, noise-free ambiance throughout the experiment. To ensure ergonomic comfort, the table and chairs were set at appropriate heights, maintaining a distance of 60 cm between the participants’ eyes and the monitor.

Three different stimulus tasks each with target item numbers of 8–16 were designed, totaling 27. In the experiment, participants were asked to try to imagine being in a time-pressured context and perform a single visual search task according to the systematic search strategy to find out the number of targets in the search content. Upon completion of the search, self-assessment of memory load was performed to determine whether search performance was affected by memory load. Before starting each session, participants ensured that they were in good mental health. The order of the tasks was randomized. If a search result was incorrect, it was not counted in the results and the experiment was repeated. The results showed that of all the negative ratings, all but one was located at “10,” while the others were located at “14,” “15,” and “16.” Therefore, the number of target items for each task element in the experiment was controlled to be “13” or less.

Participants were instructed to imagine being under time pressure and to continuously perform visual search tasks using a systematic search strategy. The stimulus content consisted of several consecutive tasks as described above. Participants were told to raise their hand to signal the termination of the experiment if they experienced visual discomfort or fatigue. The experimenter recorded the time. Before starting each session, participants were required to ensure that they were in good spirits. Before each session, participants’ mental state was ensured to be in good condition. The average duration of the 15 experiments was calculated to be 4 min and 17 s. For the formal experiment, the duration of each session was set to 4 min.

**FIGURE 2:**
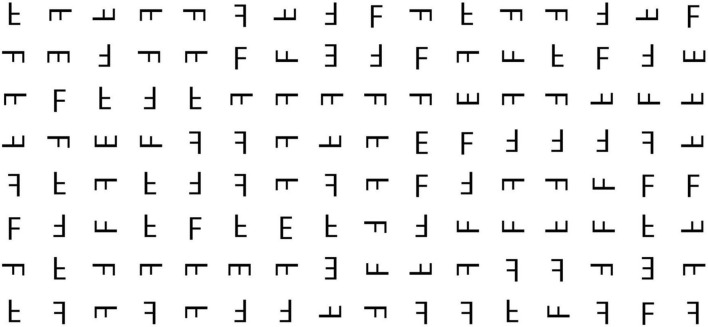
Example of the visual search task.

To regulate additional factors’ impact, like fatigue and cognitive load, each image contained no more than 13 “E” and prevented adjacent “E” occurrences. Wolfe et al. (2016) found that 25%–33% of targets are often left uncollected when moving. Therefore, it is important to avoid having two adjacent “E” characters in both horizontal and vertical directions. Multiple distinct images were crafted to function as stimuli for the visual search task (refer to Figure 2).

### 3.3 Experimental design

This study adopted a within-subjects design, employing clock timing as the independent variable and visual search performance as the dependent variable. The study has two phases, three experiments in each phase, totaling six experiments, which are E1, E2, E3, E4, E5, and E6. Each experiment consists of multiple search tasks. The different experiments are distinguished in the clock timing area of the search task interface. The E1 experiment search task interface has no clock display and performs a 4-min visual search task. Obviously, participants completed multiple search task components in the experiment. The E2 experiment search task interface is topped by a clock, starting at 00:00 and ending at 04:00. The clock continued during the participants’ execution of the search task until the experiment cut off at 4-min. The E3 experiment meter was the countdown clock, starting at 04:00 and ending at 00:00. Each experiment in the second phase still consisted of multiple search tasks, with a countdown clock at the top of the search task screen, but the countdown clock started independently for each task, meaning that the countdown clock resumed its initial value for each search task completed. The E4 experiment presented timing durations that were long time constraint. The E5 experiment presented timing durations that were medium time constraint. The E6 experiment presented timing durations that were short time constraint. The three represent low time pressure, medium time pressure, and high time pressure, respectively. It is worth noting that the clock timing duration was different for each participant, and its duration was derived from the experimental data of the first phase. The clock timing content of the different experiments is illustrated in Table 1.

**TABLE 1:** Clock timing presentation formats.

Experimental phase	Experiment number	Clock timing
First phase	E1	No presentation
	E2	Timer, starts at 00:00 and ends at 04:00
	E3	Countdown timer, starts at 04:00 and ends at 00:00
Second phase	E4	Long time constraint clock countdown (low time pressure)
	E5	Medium time constraint clock countdown (medium time pressure)
	E6	Short time constraint clock countdown (high time pressure)

To minimize the sequence’s impact on outcomes, participants were randomly assigned to three groups, each comprising an equal number of participants. The sequence of experiments for each group is outlined in Table 2. The total number of participants was a multiple of 3, ensuring uniformity across the groups.

**TABLE 2:** Experimental sequence.

Groups	First phase experiment			Second phase experiment		
Sequence for group 1	E1	E2	E3	E6	E4	E5
Sequence for group 2	E2	E3	E1	E5	E6	E4
Sequence for group 3	E3	E1	E2	E4	E5	E6

The experimental platform was developed using PsychoPy software, incorporating a timer scripted in Python. The visual search task interface was seamlessly integrated into the platform. Participants were required to identify the count of letter “E” occurrences in each task image and input the corresponding quantity using the keyboard.

### 3.4 Participants

Twenty-one undergraduate students from a specific university participated in this study. The other requirements for selecting participants were the same as those in the pilot experiment, except that the five participants from the pilot experiment were not included.

### 3.5 Equipment and environment

The experimental equipment and environment were the same as those used in the pilot experiment.

## 4 The first phase experiment

### 4.1 Experimental procedure

**FIGURE 3:**
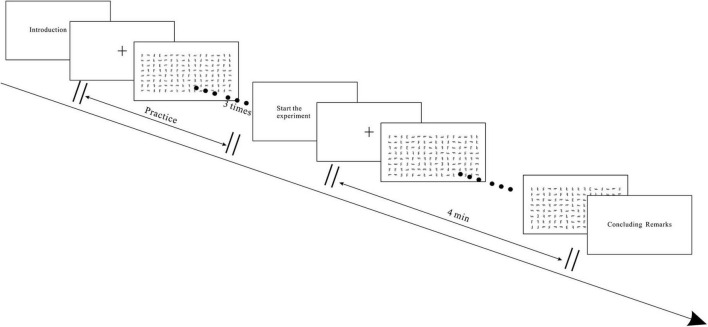
Single experiment procedure.

Before the commencement of the experiment, the experimenter ensured the proper functioning of the experimental equipment and briefed the participants on the experimental context and task. Participants were instructed to envision themselves engaging in a visual search task under emergency conditions, where both speed and accuracy were critical evaluation criteria. However, they were not informed about the presence of the timing interface or any related details. Participants were required to execute the visual search task using a systematic search paradigm. Before each experimental set, participants underwent three practice tasks to simulate searches. In the practice tasks, there is no clock at the top of the interface. Following the practice tasks, the formal experiment commenced. The experiment started with a brief instruction displayed on the screen, elucidating the content of the experiment. Participants confirmed their readiness by pressing any key on the keyboard. A fixation point, represented by a “+,” appeared at the center of the screen for 1,000 ms, succeeded by the presentation of the task image. Participants search for the letter “E” in the image and determine its quantity, then input the corresponding number using the keyboard. After each task, they pressed the “space bar” to proceed to the subsequent task, continuing until the conclusion of the 4-min experiment. The experimental procedure is depicted in Figure 3. Following each set of experiments, participants had a 2-min break before the next set. Upon completing the three experiments in the first phase, participants filled out a subjective evaluation questionnaire, detailed in Table 3.

**TABLE 3:** Subjective evaluation questionnaire.

Question	Answer
Q1: Fatigue level after the experiment	Experiment 1: ○1 ○2 ○3 ○4 ○5 Experiment 2: ○1 ○2 ○3 ○4 ○5 Experiment 3: ○1 ○2 ○3 ○4 ○5 *(1–5, with 1 being the lowest level of fatigue and 5 being the highest)*
Q2: Whether you noticed the clock timing in experiment () and experiment ()	○ Noticed and checked frequently ○ Noticed but checked occasionally ○ Did not notice

### 4.2 Data analysis

The PsychoPy software recorded participants’ response details and time, enabling the calculation of RT and accuracy for each task. The participant’s single RT was the time between the presentation of the search task and the keystroke response. Subsequently, SPSS software was used to conduct a normality test on the RTs of each participant in the experiment. The test outcomes indicated that for E1, Participant 7 exhibited a skewness value of 2.154 and a kurtosis value of 5.329, both surpassing 1.96, indicating non-normal distribution. However, all other data met the normal distribution criteria. To address outliers, the “3σ rule” was applied, effectively normalizing the data for this particular experiment.

**FIGURE 4:**
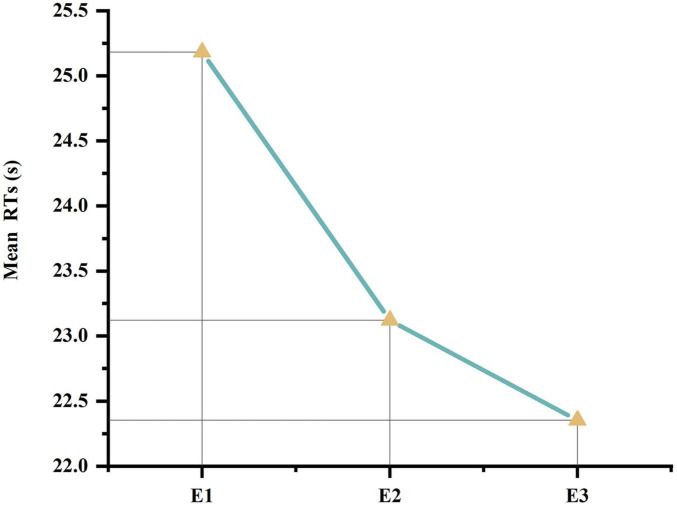
Mean RTs line chart.

The mean RT of each participant in each experiment was calculated, and a one-way analysis of variance (ANOVA) was performed on the mean RTs of participants in the three experiments. The results revealed *F*(2,60) = 4.588, *P* = 0.014, indicating a statistically significant in the mean RTs under different timing conditions (*P* < 0.05). *Post-hoc* tests indicated a statistically significant in the mean RTs between the no-timing and timing conditions (*P* = 0.038, *P* < 0.05), a highly statistically significant between the no-timing and countdown conditions (*P* = 0.005, *P* < 0.01), and not statistically significant between the timing and countdown conditions (*P* = 0.420, *P* > 0.05). Specific results are presented in Table 4. The mean RTs graphs for different experiments are illustrated in Figure 4.

**TABLE 4:** Mean RTs of different experiments (*M* ± SD).

	E1	E2	E3	*F*
Mean RTs	25.616 ± 3.089	23.561 ± 3.226	22.775 ± 3.100	4.588*

*P < 0.05.

Analysis of variance was conducted on the accuracy rates of E1, E2, and E3. The results showed *F*(2,60) = 0.146, *P* = 0.865, indicating no significant in accuracy rates under different timing conditions. Furthermore, a scoring system was used for each search task, where correct responses was assigned a value of 1 and incorrect responses was assigned a value of 2. Subsequently, a correlation analysis was performed between RT and accuracy. The calculated Kendall correlation coefficient between the two was *R* = 0.11, *P* = 0.914, with *P* > 0.05, suggesting a lack of correlation between accuracy and speed.

**FIGURE 5:**
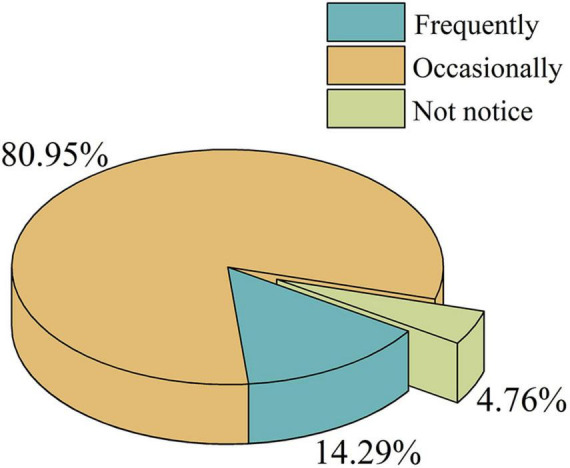
Pie chart of clock attention degree.

The questionnaire results were analyzed, and the mean fatigue index for each experiment of participants was calculated, as presented in Table 5. The participants’ level of attention to the timing of E2 and E3 was assessed, and a pie chart was created, as illustrated in Figure 5.

**TABLE 5:** Fatigue questionnaire statistics.

	E1	E2	E3
Fatigue index	1.238	1.190	1.286

**FIGURE 6:**
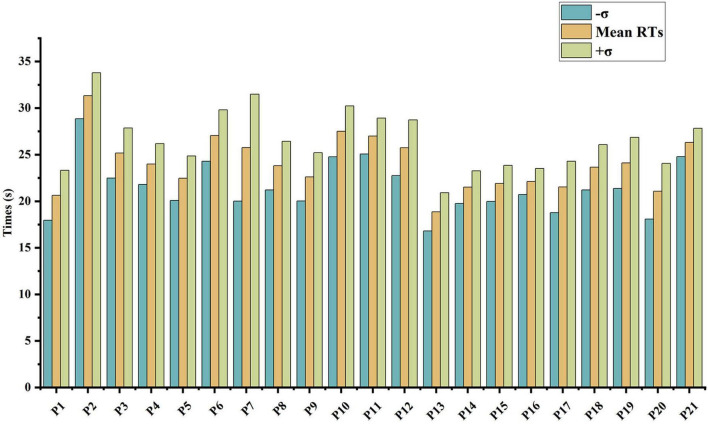
Bar graph of participant RTs.

Furthermore, the number of correct search tasks and their corresponding RTs were compiled for each participant. The mean RT for correctly performed tasks by participant *i* was denoted as $\bar{t_{i}}$, and the standard deviation was calculated as σ_*i*_. Based on this, three different time intervals, $\bar{t_{i}}+\sigma_{i}$,$\bar{t_{i}}$,$\bar{t_{i}}$-σ_*i*_ were used as the starting points for the countdown in the second phase experiments (E4, E5, and E6), representing low, medium, and high time pressure, respectively. The distribution of participants’ RTs is shown in Figure 6.

## 5 The second phase experiment

### 5.1 Experimental procedure

The experimental protocol in the second phase was similar to that for the first phase. Participants were instructed to maintain the same search strategy employed in the first phase. Additionally, they were informed about the presence of a countdown timer at the top of the search interface, necessitating the completion of each search task within a specified time range. Failure to complete a task within the designated time caused the task content to disappear, rendering the screen gray. Participants were still required to input the completed search content and press the spacebar to move to the next search. Each experiment lasted 4 min, followed by a several-minute break before the subsequent experiment. A total of three experiments were conducted. The clock counts for the three experiments are long time constraint clock countdown, medium time constraint clock countdown and short time constraint clock countdown. The order of experiments followed the order of experiments in Table 2 species. Following the completion of the second phase’s three experiments, participants filled out a subjective evaluation questionnaire identical to the fatigue questionnaire in the first phase.

### 5.2 Data analysis

The data processing method in the second phase experiment was the same as that in the first phase. The mean RT for each participant was calculated, and a normality test was conducted. The results showed that all data were normally distributed.

The mean RTs for participants in different experiments were calculated, and ANOVA was performed. The results indicated *F*(2,60) = 7.564, with *P* < 0.05, showing a statistically significant in mean RTs across the experiments. *Post-hoc* tests revealed not statistically significant in mean RT between E4 and E5 (*P* = 0.165, *P* > 0.05), a highly statistically significant between E4 and E6 (*P* < 0.001, corrected *P* < 0.0167, using Bonferroni correction), and a statistically significant between E5 and E6 (*P* = 0.018, corrected *P* < 0.0167). The mean values for E4, E5, and E6 were 22.261, 21.120, and 19.336, respectively. As the time constraints decreased and the time pressure intensified from E4 to E6, participants’ search speed increased, leading to a reduction in mean RTs.

**FIGURE 7:**
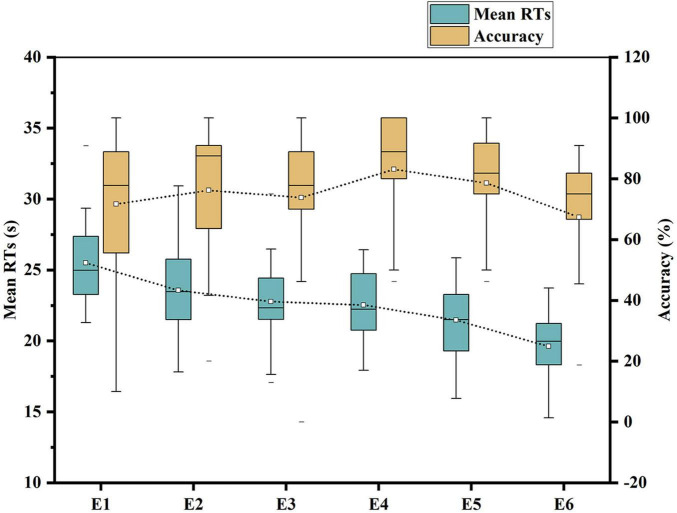
Box plot of mean RTs and accuracy.

Concerning the accuracy of visual search tasks in each experiment, ANOVA was conducted on the accuracy rates of the three experiments. The results indicated *F*(2,60) = 4.315, with *P* < 0.05, demonstrating a statistically significant in accuracy rates. *Post-hoc* tests revealed not statistically significant in accuracy between E4 and E5 (*P* = 0.415, *P* > 0.05), a highly statistically significant between E4 and E6 (*P* = 0.006, corrected *P* < 0.0167), and a statistically significant between E5 and E6 (*P* = 0.046, corrected *P* < 0.0167). Detailed results are presented in Table 6. Box plots illustrating the mean RTs and accuracy rates for the six experiments are shown in Figure 7.

**TABLE 6:** Accuracy rates in different experiments (*M* ± SD).

	E4	E5	E6	F
Accuracy rate	0.831 ± 0.168	0.786 ± 0.170	0.674 ± 0.197	4.315*

*P < 0.05.

**FIGURE 8:**
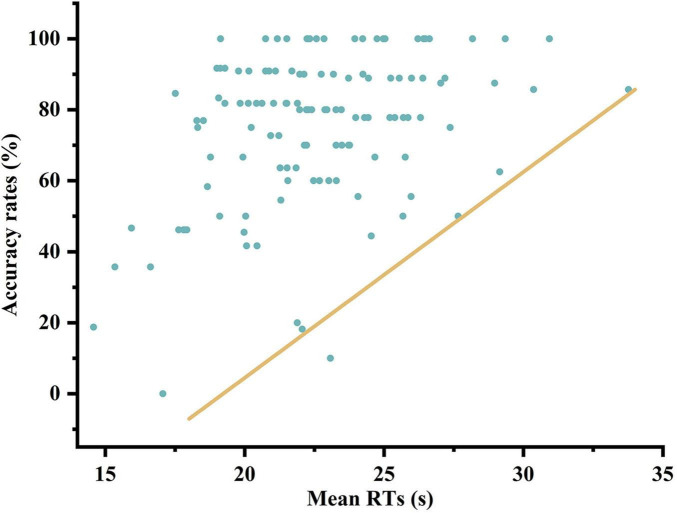
Scatter plot of mean RT and accuracy rate.

The accuracy of each search task was assigned values, where correct responses were assigned 1, and incorrect responses were assigned 2. Subsequently, a correlation analysis was conducted between mean RTs and accuracy, resulting in a Kendall correlation coefficient of *R* = −0.220, *P* < 0.01, indicating the presence of a correlation between mean RTs and accuracy. The mean RT and accuracy rate of each participant in each experiment during the two phases were compiled. Figure 8 is a scatterplot depicting the relationship between the mean RTs and accuracy rates for all experiments.

**FIGURE 9:**
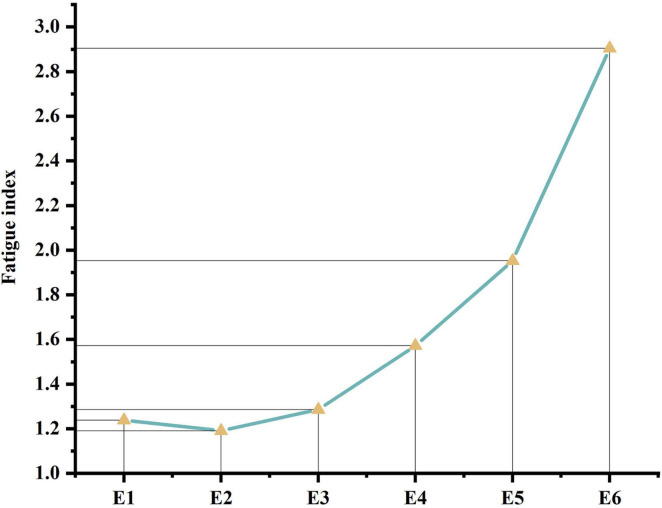
Fatigue index line graph.

In response to the questionnaire results, the average fatigue index was computed for each experiment involving the participants. A line graph depicting the fatigue index across the six experiments is presented in Figure 9.

## 6 Discussion

### 6.1 Impact of timing methods on search efficiency

Based on the data from the first phase of the experiment, there was a significant difference in the mean RT between the conditions with no clock display and those with clock timing. RT represents search speed, where shorter RTs indicate faster search speeds. The results indicated that the search speed was faster under the clock timing condition than without clock timing. Questionnaire results revealed that 95.24% of the participants were aware of the presence of the clock, which impacted their search speed. This may be attributed to the heightened sensitivity to time perception under timed conditions, leading to a certain degree of time pressure and accelerating the search speed. This provides insights for the design of visual search task interfaces, suggesting that displaying timing information can enhance search efficiency. In addition, there was not statistically significant in the mean RT between the clock timing and countdown clock conditions, but the mean RT in the countdown condition was slightly lower than that in the clock timing condition. This finding also supports Crescenzi et al.’s (2021) viewpoint that “users’ search behaviors did not present significant.” From an individual perspective, there were variations in the speed of visual search tasks among different individuals, which is consistent with Clarke et al.’s (2022) viewpoint that significant individual differences exist in visual search tasks.

In the first phase of the experiment, not statistically significant were observed in the accuracy under the three conditions. E2 (clock timing of timer) had slightly higher accuracy (including mean, upper quartile, etc.) than the other two groups. The accuracy in E1 (clock timing of no presentation) and E3 (clock timing of countdown timer) was relatively consistent. An analysis of the correlation between speed and accuracy revealed no significant relationship between the two. This contradicts the conclusion drawn by Van der Vegt et al. (2020) that faster speeds lead to higher error rates. Furthermore, Ren et al.’s (2022) study suggested that a well-arranged rest schedule is conducive to relieving visual fatigue and improving performance.

On comprehensive analysis, although the participants accelerated their search speed when they were aware of the timing, the total time constraint was long, far exceeding the time required for a single task. Therefore, the participants could freely control the time required for a single search task within the relatively long time constraint, enabling them to maintain a higher accuracy effectively. For instance, when the participants perceived that the current search task took longer or resulted in a search error, they would hasten their search speed in the next task. The long time constraint provided the participants with more flexibility, enabling them to allocate time for each task more effectively, thereby reducing time pressure and lowering the error rate. It also suggests that there is a need for research on visual search task performance under shorter time constraints, and that a second phase of experiments is in order. From the subjective fatigue level of the participants, the fatigue level under the three conditions was relatively low and did not show significant. This suggests that during the three experiments in the first phase, the participants did not experience noticeable fatigue, and fatigue did not significantly impact search performance.

### 6.2 Impact of different time constraints on search efficiency

The second phase of the experiment involved completing visual search tasks within shorter time constraints. Regarding search speed, it is evident that the shorter the time constraint, the shorter the mean RT, resulting in faster search speeds. The actual mean RT data supported this observation. Thus, we mainly focused on the differences in accuracy in visual search tasks under different time constraints and discussed their causes.

The results showed that there was a statistically significant in the percentage of correctness in E6 (the short-duration timing group) group from that of E4 (the long-duration), and E5 (the medium-duration) groups. From the standpoint of mean accuracy, an increase in leniency within the countdown time constraints corresponded to an improvement in accuracy. Defining the different time constraints as different pressure levels based on methods such as adding or subtracting 1 SD from the mean of each individual’s single RT (Liao, 2023), we found that high time pressure was not conducive to improving accuracy. Additionally, we found a correlation between single RT and accuracy, which is consistent with Mccarley’s (2009) assertion that faster searches are more prone to errors and supporting Teevan et al.’s (2013) concept of “slow search.” However, there was not statistically significant in accuracy between E4 (the long time constraint group) and E5 (the medium time constraint group). Nevertheless, the accuracy in the medium time constraint group was lower than that in the long time constraint group in terms of mean, median, upper and lower quartiles. Thus, within a reasonable time limit range, the difference in accuracy was not significant. However, once the time limit exceeded this range, it would significantly impact accuracy. It is particularly noteworthy that in the second phase experiment, the individual time constraints were determined based on each participant’s correct search task time in the first phase, thus mitigating the impact of individual differences on the experimental results.

In terms of fatigue, participants’ subjective fatigue deepened and the rate of fatigue accelerated as the time limit was shortened. Previous studies have suggested that visual search accuracy is adversely affected under fatigued conditions (Grady et al., 2022). The experimental results similarly demonstrated a trend of intensified fatigue and decreased accuracy as the time constraints shortened. Therefore, it is evident that performing visual search tasks for prolonged periods under shorter time constraints is not advisable.

### 6.3 Comparison of the two phase experiments

Comparison of the mean RTs for the two phases of the experiment revealed that there was almost not statistically significant between the second phase and the first phase. However, a statistically significant was observed between the medium time constraint group and the short time constraint group in the second phase compared to the first. This demonstrates the substantial influence of time constraints on accelerating search speed, particularly when employing clock timing with specific time constraint restrictions, thereby improving visual search speed. Prior to the start of all experiments, participants were asked to try to imagine being in a time-pressured environment to carry out a visual search task. Previous studies by Alqahtani et al. (2016) and Irwin et al. (2013) exploring the impact of time pressure by prompting participants to operate within such contexts highlighted the subjective nature of generating time pressure. Therefore, in experiments conducted under a time pressure background, using the form of time constraint can create a certain degree of time pressure on the participants.

Taking into account the comprehensive results of the two phase experiments, utilizing participants’ mean RT as the time constraint, i.e., medium time pressure, can offers a better equilibrium between search speed and accuracy, enhancing the overall performance in visual search tasks. Comparing accuracy between the two phases revealed higher accuracy within the long and medium time constraint groups compared to the initial phase. Moreover, the stability of accuracy was higher in the second phase of the experiment than in the first phase. Search speed during the second phase of the experiment surpassed that of the first phase. Typically, accelerated search speed correlates with reduced accuracy. Consequently, accuracy during the second phase was expected to be lower than the first. However, this discrepancy suggests other factors influencing accuracy, potentially proficiency-related, as prior studies have suggested (Boot et al., 2009). Although we set a long time interval between the two phases of the experiment, proficiency might still impact performance. Therefore, the effect of proficiency on visual search performance under time pressure conditions requires further exploration.

The scatter plot illustrating mean RT and accuracy shows that most data points lie above the line on the graph. Mean RT, which reflects speed, reveals few data points in the low-speed, low-accuracy range and even fewer in the high-speed, high-accuracy range. This indicates that slower searches generally yield higher accuracy, while increased speed is associated with reduced accuracy. Notably, this trend remains consistent across various factors, including timing conditions and individual differences among participants.

When comparing fatigue levels between two phases, the tasks in the first phase, within a 4-min time constraint, did not induce significant fatigue. In contrast, during the second phase, where participants performed tasks of the same total duration as in the first phase, clear fatigue emerged across all three experiments, notably stronger than in the first phase. This indicates that fatigue onset was accelerated under short-duration countdown constraints. The interplay between time constraints and fatigue levels affects accuracy.

## 7 Summary

Conducting VDT visual search tasks under time constraints has broad applications. Time constraint exhibits a dual nature, and clock timing stands as a crucial component of the VDT visual search interface. Investigating the impact of clock timing on the performance of visual search tasks holds significant significance. This study explored the impact of various forms of clock timing and time constraints on VDT visual search performance, particularly probing how clock timing influences search speed, accuracy, and related aspects.

The results of this study can be summarized as follows: (1) In scenarios where the time constraint with clock timing significantly exceeds the execution time of a single task, it improves search speed without compromising accuracy compared to scenarios without clock displays. Notably, there were no substantial differences observed in search speed and accuracy between clock timing and countdown clock timing. (2) In scenarios where the time constraint with clock timing closely matches the duration of a single task, shorter constraints lead to faster search speeds but also accompany decreased accuracy and heightened levels of fatigue. By using participants’ mean RTs as the medium time constraint and employing ±1 SD as long and short constraints, respectively, higher accuracy was achieved in medium and long time constraints across all three conditions. Considering search speed, accuracy, and fatigue, embracing medium and long time constraints augments overall visual search performance. (3) In visual search tasks, there is a noticeable correlation between search speed and accuracy—usually, higher search speed correlates with lower accuracy. Therefore, designing clock timing in alignment with task attributes and content is pivotal for enhancing search performance. Tasks necessitating high accuracy may benefit from longer time constraints, whereas those requiring speed may opt for medium constraints.

These findings offer insights for refining timing in visual search task interfaces under time pressure. Tailoring timing methods to task attributes holds promise for enhancing visual search performance. Future research endeavors could explore the impact of fatigue levels under various time constraint restrictions and devise pragmatic rest strategies.

## Data availability statement

The raw data supporting the conclusions of this article will be made available by the authors, without undue reservation.

## Ethics statement

The studies involving humans were approved by the Ethics Committee of the School of Art and Design at North China Institute of Science and Technology. The studies were conducted in accordance with the local legislation and institutional requirements. The participants provided their written informed consent to participate in this study.

## Author contributions

JBH: Conceptualization, Data curation, Investigation, Methodology, Writing – original draft, Writing – review & editing. QC: Investigation, Supervision, Visualization, Writing – original draft. DL: Validation, Visualization, Writing – review & editing. JKH: Data curation, Investigation, Writing – original draft.
